# Generating vectorial optical fields via surface-wave-excited complex-amplitude metasurfaces

**DOI:** 10.1038/s41377-026-02334-1

**Published:** 2026-05-27

**Authors:** Xiangyu Jin, Yu He, Jianru Li, Xiaoya Nie, Shuai Du, Yufei Song, Haoyu Luo, Muhan Liu, Shaojie Ma, Qiong He, Lei Zhou, Zhuo Wang, Shulin Sun

**Affiliations:** 1 Shanghai Engineering Research Centre of Ultra Precisio Optical Manufacturing, Department of Optical Science and Engineering, College of Future Information Technology, Fudan University, Shanghai, China; 2 State Key Laboratory of Surface Physics, Key Laboratory of Micro and Nano Photonic Structures (Ministry of Education) and Department of Physics, Fudan University, Shanghai, China; 3 Shanghai Key Laboratory of Metasurfaces for Light Manipulation, Shanghai, China; 4 Shanghai Frontiers Science Research Base of Intelligent Optoelectronics and Perception, Institute of Optoelectronics, Fudan University, Shanghai, China

**Keywords:** Metamaterials, Nanophotonics and plasmonics

## Abstract

On-chip photonic systems capable of efficiently generating pre-designed vectorial optical fields (VOFs) are highly desired in integrated photonics, but traditional devices are bulky and lack flexible control capabilities. Although ultra-compact metasurfaces (MSs) have exhibited powerful light-manipulation capabilities, they typically work under propagating-wave excitations and/or rely on only phase modulations to control light beams. Here, a general strategy is proposed to design MSs that, under surface wave (SW) excitations, can independently control the *amplitudes* and *phases* of locally scattered waves with two orthogonal polarizations, thus enabling efficient generation of pre-designed VOFs. As a benchmark test, a *complex amplitude* MS was constructed to experimentally demonstrate the generation of two directional beams exhibiting orthogonal polarizations and arbitrarily pre-designed intensities, under excitation of SW at 0.4 THz. Next, another MS was experimentally demonstrated to generate two focal points in the far field with distinct intensities, under the same THz SW excitation. Finally, based on a modified Gerchberg-Saxton (GS) algorithm incorporating both amplitude and phase modulations, a series of THz SW-excited *complex amplitude* MSs were designed and fabricated, and were experimentally demonstrated to respectively generate pre-designed *scalar* and *vectorial* holographic images in the far field, exhibiting much improved qualities and flexibility than those generated by their phase-only counterparts. The present work establishes a novel on-chip platform to generate complex vectorial fields, paving the way for many applications in integrated optics such as encrypted holography, and augmented reality.

## Abstract (web summary)

We propose a design strategy for surface-wave-excited complex-amplitude metasurfaces that independently control the amplitude, phase, and polarization of locally scattered waves and thus generate predefined vector optical fields.

## Graphical abstract

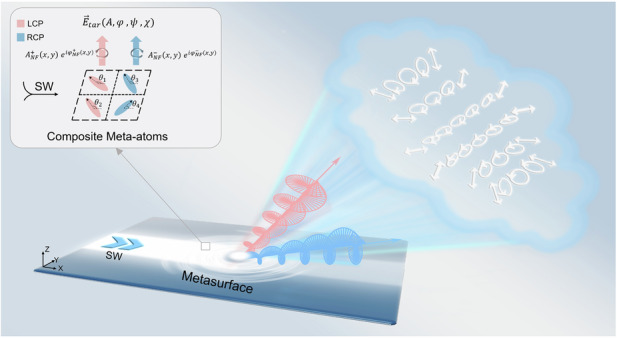

## Introduction

On-chip photonic systems capable of efficiently generating pre-designed vectorial optical fields (VOFs) are highly desired for integrated-photonics research and applications^1,2^. Unlike traditional scalar fields exhibiting uniform polarization distributions, VOFs leverage the additional degree of freedom provided by inhomogeneous polarizations, enabling a broader range of applications, including super-resolution microscopy^3,4^, optical trapping, and manipulation^5,6^. Conventional methods for generating VOFs, however, often involve complicated experimental setups composed of optical modulators, waveplates, spatial light modulators or other devices^7,8^, being unfavorable for optical integrations.

Metasurfaces (MSs) are planar metamaterials composed of subwavelength microstructures with precisely engineered light-scattering properties (including amplitude, phase and polarization), which have demonstrated unprecedented capabilities in controlling electromagnetic (EM) waves^9,10^. Various fascinating wavefront-manipulation effects have been achieved using MSs exhibiting different phase distributions, such as anomalous reflection and refraction^9,11,12^, surface wave (SW) excitation^10,13^, beam focusing^14–17^, meta-holography^18–24^, and many others^25–29^. Later, based on subwavelength meta-atoms that can control both phase and polarization of locally scattered waves, numerous MSs were realized to generate complex VOFs in the desired manner^30–33^, significantly expanding capabilities to manipulate EM waves. However, these MSs usually work under propagating-wave (PW) excitations, which are still inconvenient for optical integrations.

Recently, on-chip meta-devices working under SW excitations have gained intensive attention due to their superior compatibilities with integrated photonic applications compared with their PW-excited counterparts. With propagation phases of exciting SWs taken into account, various MSs were designed to decouple on-chip SWs into free-space PWs with arbitrary wavefronts, such as unidirectional radiation^34^, far-field focusing^35–37^, and holography^38,39^, etc. Recently, SW-excited MSs capable of generating VOFs in the far field have also been realized^40,41^. However, most on-chip MSs realized so far rely solely on spatial phase modulations to engineer the wavefronts of decoupled light beams. Without amplitude modulations, beams generated by such meta-devices exhibit restricted properties, especially concerning the quality of holographic reconstruction. Although a few SW-excited meta-devices leveraging both *amplitude* and *phase* modulation were recently proposed to achieve versatile functionalities simultaneously^42–45^, SW-excited MSs that can generate arbitrary VOFs through *precise*, *continuous* and *independent* modulation of amplitude, phase, and polarization have been rarely reported.

**Fig. 1:**
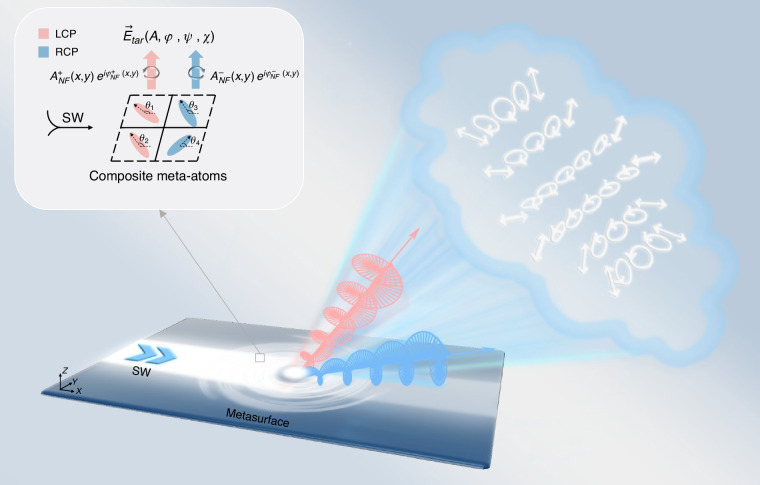
Diagram of the composite MSs for generating complex-amplitude VOFs under surface wave excitation. The building block is composed of 2 × 2 meta-atoms that can freely modulate the amplitude, phase and polarization of the radiation field at the subwavelength scale

Here, a general strategy is proposed for designing on-chip MSs that can generate tailored VOFs under SW excitations, based on *full-parameters* controls over locally scattered waves in terms of amplitude, phase, and polarization (see Fig. 1). Such modulation is enabled by utilizing composite meta-atoms consisting of **2** × **2** resonators with carefully tailored orientations and structures. To validate this concept, four terahertz (THz) meta-devices were designed, fabricated and their functionalities were experimentally characterized under SW excitations. The first meta-device can generate two circularly polarized (CP) directional beams exhibiting opposite helicities and pre-designed field intensities, while the second one can generate two linearly polarized beams focused at different far-field positions exhibiting pre-determined intensities. Finally, using an improved iterative Gerchberg-Saxton (GS) algorithm that simultaneously incorporates both amplitude and phase modulations, the last two meta-devices were designed and fabricated, and it was experimentally demonstrated to generate *scalar* and *vectorial* holographic images with qualities significantly superior to those achieved by phase-only (PO) counterparts, respectively. Full-wave simulations were performed on these devices, showing excellent agreement with experimental results.

## Results

### Fundamental principle

**Fig. 2:**
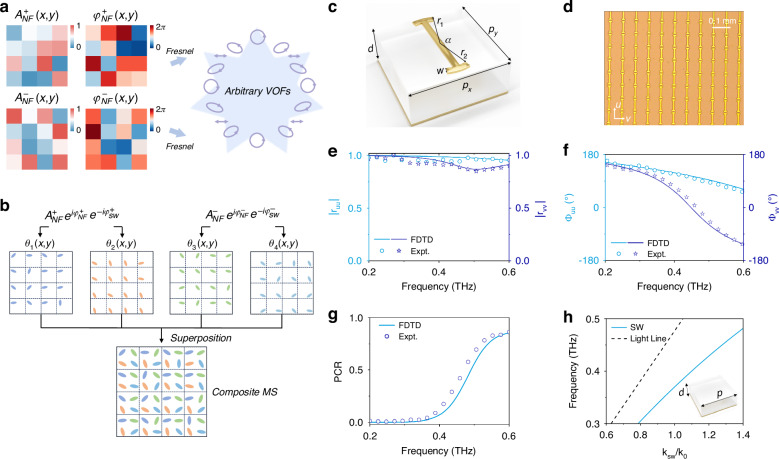
Principle of generating desired VOFs distributions by SW metasurface and characterization of the PB meta-atoms and plasmonic metal. **a** The amplitude and phase of two orthogonal polarization states (LCP and RCP) for generating the desired VOFs. **b** The design strategy of the on-chip composite MS constructed by the PB meta-atoms with individual rotation angle distributions for achieving the complex fields shown in **a**. **c**, **d** Schematic illustration and sample image of the designed meta-atom. **e**-**g** Calculated and measured reflection phase (Φ_*uu*_ and Φ_*vv*_), reflection amplitude (${{|r}}_{{uu}}|$ and ${{|r}}_{{vv}}|$) and polarization conversion ratio (PCR) spectra of the periodic PB meta-atoms illuminated by the terahertz light linearly polarized along the two principal axes, i.e., u and v axes. **h** Calculated dispersion relation of the eigen SW modes supported by the designed plasmonic metal (see the inset)

The near-field (NF) source required for generating a VOF was first discussed. An on-chip MS was designed to realize an *arbitrary* VOF in the far field. Under a SW beam excitation, it can generate a field distribution on the source *xy*-plane exhibiting the following *general* form:

1 $${\vec{E}}_{{NF}}\left({\boldsymbol{r}}\right)={\vec{E}}_{{NF}}^{+}\left({\boldsymbol{r}}\right)+{\vec{E}}_{{NF}}^{-}\left({\boldsymbol{r}}\right)={A}_{{NF}}^{+}\left(x,y\right){e}^{i{\varphi }_{{NF}}^{+}\left(x,y\right)}+{A}_{{NF}}^{-}\left(x,y\right){e}^{i{\varphi }_{{NF}}^{-}\left(x,y\right)}$$

where ${A}_{{NF}}^{+}\left(x,y\right)$ and ${A}_{{NF}}^{-}\left(x,y\right)$ denote the amplitude distributions of LCP ($\sigma =+1$) and RCP ($\sigma =-1$) components emitted by the SW excited MS, respectively, while ${\varphi }_{{NF}}^{+}\left(x,y\right)$ and ${\varphi }_{{NF}}^{-}\left(x,y\right)$ represent their corresponding local phase distributions. Equation (1) indicates that such a MS enables simultaneous modulation of the local amplitude, phase, and polarization of the electric field, which is commonly referred to as a complex-amplitude (CA) MS. According to Huygens’ principle, these NF distributions can be tailored to generate different VOFs in the far-field, exhibiting pre-designed wavefronts and polarization distributions, as schematically shown in Fig. 2a.

Next, the strategy for freely modulating the phase profile of the NF source based on a SW-excited MS was discussed. First of all, the previously developed Pancharatnam-Berry (PB)-based approach was recalled to design on-chip meta-devices^34,40,41^. As a SW beam interacts with the MS, waves can be decoupled to free-space due to the inverse wave-vector compensation. The phase response of radiation field at each local position can be precisely controlled by adjusting the rotation angle of each meta-atom, enabling free controls of CP light fields with polarization states $$\left[\begin{array}{c}1\\ \pm i\end{array}\right]$$. To generate a target CP beam, the phase profile ${\varphi }_{{ms}}^{\sigma }(x,y)$ required for designing a MS can be determined by:

2 $${\varphi }_{{NF}}^{\sigma }\left(x,y\right)={k}_{{sw}}x+{\varphi }_{{ms}}^{\sigma }\left(x,y\right)$$

where ${k}_{{\rm{sw}}}x$ denotes the initial phase accumulated by the incident SW propagating along the device, and ${\varphi }_{{NF}}^{\sigma }\left(x,y\right)$ denotes the phase profile of the NF source on the MS plane for generating the target beam.

However, such an approach can only enable phase modulation which is insufficient to achieve the desired goal. Next, a new strategy was developed to achieve independent modulation of the local amplitudes and phases of two orthogonal CP components. As illustrated in Fig. 2b, each supercell in the MS consisted of **4** identical PB meta-atoms with distinct rotation angles: ${\theta }_{1},{{\theta }_{2},{\theta }_{3},\theta }_{4}$, arranged in a **2** × **2** matrix. In each supercell, two meta-atoms on the left column were designed to generate the LCP field with the desired CA distribution, while the two meta-atoms on the right column were responsible for the RCP field. Considering that the NF source emitted by the MS carries both a spin-dependent PB phase, which is proportional to twice of the rotation angle of the local meta-atom (${\varphi }_{{ms}}^{\sigma }\left(x,y\right)=\sigma \cdot 2\theta$), and a spin-independent propagation phase delay of SW (${\varphi }_{{sw}}^{\sigma }={k}_{{sw}}x$), the following expressions were obtained:

3 $$\begin{array}{c}{{\vec{E}}_{{NF}}^{+}\left({\boldsymbol{r}}\right)=A}_{{NF}}^{+}\left(x,y\right){e}^{i{\varphi }_{{NF}}^{+}\left(x,y\right)}=\frac{1}{2}\left({e}^{+i2{\theta }_{1}\left(x,y\right)}+{e}^{+i2{\theta }_{2}\left(x,y\right)}\right){e}^{i{\varphi }_{{sw}}^{+}}\\ {{\vec{E}}_{{NF}}^{-}\left({\boldsymbol{r}}\right)=A}_{{NF}}^{-}\left(x,y\right){e}^{i{\varphi }_{{NF}}^{-}\left(x,y\right)}=\frac{1}{2}\left({e}^{-i2{\theta }_{3}\left(x,y\right)}+{e}^{-i2{\theta }_{4}\left(x,y\right)}\right){e}^{i{\varphi }_{{sw}}^{-}}\end{array}$$

to determine the local NF amplitudes/phases {${A}_{{NF}}^{\pm }\left(x,y\right){,\varphi }_{{NF}}^{\pm }\left(x,y\right)$} in terms of local orientation angles $\{{\theta }_{i}(x,y)$}. Now the advantages of taking such a supercell consisting of 2 × 2 meta-atoms are clear: whereas each single meta-atom can only yield phase modulation, the interference among them allows for precise controls of both amplitudes and phases of two CP components by adjusting four distinct angles $\{{\theta }_{i}\}$. Given the target {${A}_{{NF}}^{\pm }\left(x,y\right){,\varphi }_{{NF}}^{\pm }\left(x,y\right)$} distributions to realize, the corresponding {*θ*_*i*_} distributions could be retrieved by the following equations:

4 $$\begin{array}{lc}{\theta }_{1}\left(x,y\right)-{\theta }_{2}\left(x,y\right) =co{s}^{-1}\left(\left|{A}_{NF}^{+}\left(x,y\right){e}^{i{\varphi }_{NF}^{+}\left(x,y\right)}{e}^{-i{\varphi }_{sw}^{+}}\right|\right)\\ {\theta }_{1}\left(x,y\right)+{\theta }_{2}\left(x,y\right) =arg\left({A}_{NF}^{+}\left(x,y\right){e}^{i{\varphi }_{NF}^{+}\left(x,y\right)}{e}^{-i{\varphi }_{sw}^{+}}\right)\\ {\theta }_{3}\left(x,y\right)-{\theta }_{4}\left(x,y\right) =co{s}^{-1}\left(\left|{A}_{NF}^{-}\left(x,y\right){e}^{i{\varphi }_{NF}^{-}\left(x,y\right)}{e}^{-i{\varphi }_{sw}^{-}}\right|\right)\\ {\theta }_{3}\left(x,y\right)+{\theta }_{4}\left(x,y\right) =-arg\left({A}_{NF}^{-}\left(x,y\right){e}^{i{\varphi }_{NF}^{-}\left(x,y\right)}{e}^{-i{\varphi }_{sw}^{-}}\right)\end{array}$$

It is noted that {${\theta }_{1}(x,y)-{\theta }_{2}(x,y)$} and {${\theta }_{3}\left(x,y\right)-{\theta }_{4}\left(x,y\right)$} govern two amplitude distributions, $\left\{{\theta}_{1}\left(x,y\right)+{\theta}_{2}(x,y)\right\}\,{\rm{and}}\,\left\{{\theta}_{3}(x,y)+{\theta}_{4}(x,y)\right\}$ determine two phase distributions, as schematically shown in Fig. 2b.

As a final step, the retrieval of the {${A}_{{NF}}^{\pm }\left(x,y\right){,\varphi }_{{NF}}^{\pm }\left(x,y\right)$} distributions of the MS was discussed, enabling the generation of an arbitrary pre-designed complex VOF. Based on angular spectrum theory and the paraxial approximation, the target VOF was decomposed into two spin components, each of which could be generated by a spin-polarized NF source. Based on Fresnel’s diffraction theory, the VOF was expressed in terms of {${A}_{{NF}}^{\pm }\left(x,y\right){,\varphi }_{{NF}}^{\pm }\left(x,y\right)$} distributions of the NF source as:

5 $${\vec{E}}_{{tar}}\left({\boldsymbol{r}}\right)={\vec{E}}_{{tar}}^{+}\left({\boldsymbol{r}}\right)+{\vec{E}}_{{tar}}^{-}\left({\boldsymbol{r}}\right)=\frac{\exp \left({ikd}\right)}{i\lambda d}{F}^{-1}\left\{\left[F\left[{A}_{NF}^{+}\left({\boldsymbol{r}}\right){e}^{i{\varphi }_{NF}^{+}\left(x,y\right)}\right]+F\left[{A}_{NF}^{-}\left({\boldsymbol{r}}\right){e}^{i{\varphi }_{NF}^{-}\left(x,y\right)}\right]\right]\cdot F\left[\exp \left(\frac{jk}{2d}\left({x}^{2}+{y}^{2}\right)\right)\right]\right\}$$

where ${\vec{E}}_{{tar}}^{+}\left({\boldsymbol{r}}\right)$ and ${\vec{E}}_{{tar}}^{-}\left({\boldsymbol{r}}\right)$ denote the CA field distributions for LCP and RCP components on the target plane, and *F* and *F*^−1^ represent the Fourier and inverse Fourier transform, respectively. Therefore, the NF source distributions {${A}_{{NF}}^{\pm }\left(x,y\right){,\varphi }_{{NF}}^{\pm }\left(x,y\right)$} of the MS were determined from ${\vec{E}}_{{tar}}^{+}\left({\boldsymbol{r}}\right)$ and ${\vec{E}}_{{tar}}^{-}\left({\boldsymbol{r}}\right)$ known from the target VOF, according to the standard iterative procedures based on Eq. (5). With the desired amplitude and phase distributions known, the corresponding $\{{\theta }_{i}\}$ distributions were derived from Eq. (4), implying the completion of the on-chip MS design.

### Design of meta-atom and plasmonic-metal

While the strategy works for arbitrary frequencies, the THz frequency regime was chosen here to demonstrate the concept. As shown in Fig. 2c, the meta-atom was designed in a metal-insulator-metal (MIM) configuration, consisting of a curved I-shaped gold microstructure (*p* = 80 μm, *r*_1_ = 35 μm, *r*_2_ = 30 μm, *w* = 5 μm, *α* = 145°) and a continuous gold film, separated by a 55-μm thick quartz spacer (*ε*_*r*_ = 3.9 + 0.001*i*). It is clear that such a meta-atom exhibits mirror symmetry with their two principal axes labeled as u and v, respectively. A sample consisting of an array of such meta-atoms arranged in a square lattice was fabricated (see Fig. 2d for its optical image), and its scattering properties were characterized using a THz time-domain spectroscopy (TDS) system. Fig. 2e depicts the reflection-phases ${\varPhi }_{{uu}}$ and ${\varPhi }_{{vv}}$ of the sample illuminated by THz beams polarized along two principal axes, obtained by both finite-difference time-domain (FDTD) simulations and experimental measurements. The measured phase difference ${\varPhi }_{{uu}}-{\varPhi }_{{vv}}$ was found to increase continuously from 5° to 165° as frequency increased from 0.2 THz to 0.6 THz. Since the bottom layer gold film completely blocks the transmission channel, measured reflection amplitudes ${{|r}}_{{uu}}|$ and ${{|r}}_{{vv}}|$ of the MS are close to1, as shown in Fig. 2f. Polarization conversion ratio (PCR) of the designed meta-atom, retrieved from the measured reflection coefficients via ${\left|({r}_{{uu}}-{r}_{{vv}})/2\right|}^{2}$, is depicted in Fig. 2g as a function of frequency. According to the previous analyses^34,40,41^, the PCR of the meta-atom is proportional to the amplitude of the locally scattered wave, and the amplitude of the excitation SW decays as it flows on the MS. Therefore, an optimized PCR is needed to achieve a high device efficiency and a uniform wavefront. Here, by carefully balancing efficiency and wavefront flatness, the meta-atom was purposely designed to exhibit a low PCR (PCR≈0.1) at the target frequency 0.4 THz, as shown in Fig. 2g.

An artificial plasmonic metal supporting eigen SWs with appropriate wave vectors was also designed to serve as a platform for the injection of the excitation SW beam. A gold thin film does not support eigen SWs with the needed wavevectors in the THz regime due to the extremely large conductivity of metal. Therefore, a 55-μm thick quartz spacer (${\rm{with}}\,{{\rm{\varepsilon }}}_{r}=3.9+0.001i$) was placed on a flat gold film to form an artificial plasmonic metal. Figure 2h depicts the simulated dispersion relations of spoof SWs supported by the artificial plasmonic metal. The eigen SW system has been carefully optimized to support SW modes with wave functions matching well with those of the on-chip metasurface, thereby suppressing undesired SW scattering at the boundary between them.

Next, the generic strategy described in the section entitled “Fundamental principle” was employed to design a series of SW-excited CA MSs for generating desired far-field beams with increasing complexity, based on the designed meta-atom and artificial plasmonic metal.

### Dual-beam radiation with pre-designed intensities and polarizations by on-chip complex-amplitude metasurface

**Fig. 3:**
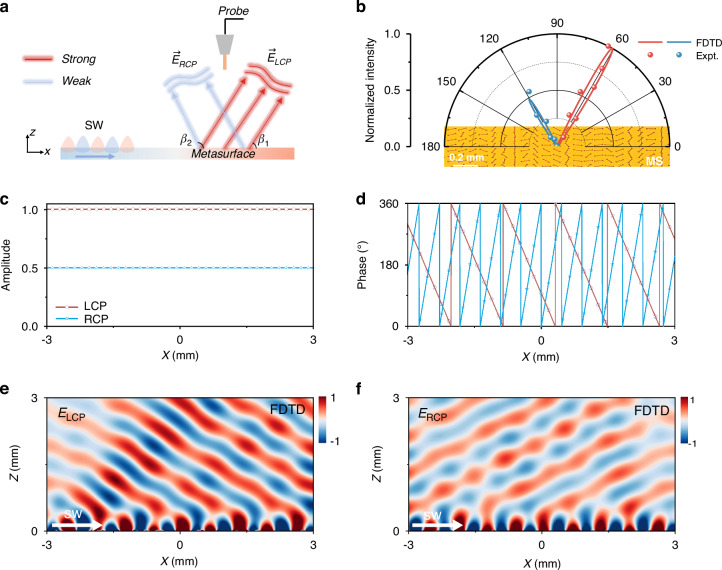
Far-field angle-resolved characterization of dual-directional PW radiation beams with designed intensity ratio and orthogonal polarizations using the composite metasurface under SW excitation. **a** Schematic illustration and measurement setup for the metadevice to generate two plane wave beams with orthogonal polarizations and different intensities decoupled to the predefined angles *β*_1 _ = 60° and *β*_2 _= 120° under the SW excitation. **b** Simulated and measured scattering field angular distribution of achieved SW-PW radiation effect by the MS. The inset shows part of the image of the fabricated MS sample. **c**, **d** Amplitude and phase profiles encoded by the MS, which are responsible for emitting two orthogonal components carrying LCP and RCP. **e**, **f** Simulated results of *E*_*lcp*_ and *E*_*rcp*_ field patterns in the *xz* plane for the composite MS under the excitation of SW at 0.4 THz

As a benchmark test, the first on-chip meta-device was designed to generate two unidirectional beams with desired radiation directions characterized by tangential wavevectors *β*_1_ and *β*_2_, as well as specific amplitudes and polarizations, under the SW excitation at 0.4 THz (see Fig. 3a). Therefore, the strategy established in the previous section was followed to retrieve the amplitude–phase distributions of the MS from the target far-field beam. In this case, numerical iterations based on Eq. (5) were not required. Instead, the amplitude-phase distributions {${A}_{{NF}}^{\pm }\left(x,y\right){,\varphi }_{{NF}}^{\pm }\left(x,y\right)$} were analytically obtained based on the generalized Snell’s law. The corresponding parameters were expressed as:

6 $$\begin{array}{lll}{A}_{{NF}}^{+}\left(x,y\right)&=&1\\ {A}_{{NF}}^{-}\left(x,y\right)&=&\frac{1}{\sqrt{2}}\\ {\varphi }_{{NF}}^{+}\left(x,y\right)&=&{k}_{0}cos{\beta }_{1}x\\ {\varphi }_{{NF}}^{-}\left(x,y\right)&=&{k}_{0}cos{\beta }_{2}x\end{array}$$

For the parameter values ${\beta }_{1}={60}^{^\circ }$ and ${\beta }_{2}={120}^{^\circ }$, the rotation angle distributions {*θ*_*i*_} of the meta-atoms were retrieved from Eq. (6) based on Eq. (4). The amplitude-phase distributions of the MS were obtained (see Fig. 3c, d). Subsequently, a sample was fabricated, consisting of identical meta-atoms designed in the previous section arranged with the retrieved {*θ*_*i*_} distributions and connected to the artificial plasmonic metal described above (see more details on fabrication in the Supplementary Information Section 1).

Experimental measurements were performed to validate the theoretical predictions. In the experiments, a SW beam at 0.4 THz was launched on the “artificial plasmonic metal” by illuminating a SW meta-coupler with a plane THz wave (details can be found in the Supplementary Information Section 1), which was subsequently injected into the MS (see Fig. 3b). A tip-based THz TDS scanning system was then utilized to detect the scattered field distribution on the *xz* plane right above the MS under SW excitation (see more details in the Supplementary Information Section 3). Next, the angular distributions of the scattered electric fields carrying LCP and RCP were then obtained by Fourier transforming the measured near-field patterns, respectively. Moreover, FDTD simulations were also employed to calculate the radiation field patterns of LCP and RCP components as shown in Fig. 3e, f. Based on the measured amplitude distributions of the two spin-polarized beams in *k*-space, the energy ratio of their radiation powers was further extracted (see Fig. 3b) to be approximately 1:0.53, in close agreement with the simulated result (1:0.49) and the designed value (1:0.5). In addition, the measured radiation directions of the two spin-polarized beams were 62° and 118°, respectively, matching well with simulation results (60.4° and 120.4°) and theoretical design (60° and 120°). Meanwhile, the working efficiency of the MS, defined as the ratio between the power carried by two radiated beams and that by the incident SW that interacted with the MS, was evaluated to be 85.81% based on numerical simulations. Although the current design demonstrated a high working efficiency, its performance could be further improved through optimizing the geometric structures and composite materials to minimize scattering and absorption losses.

### Dual-focus on-chip meta-lens with arbitrary controlled intensity and polarization

**Fig. 4:**
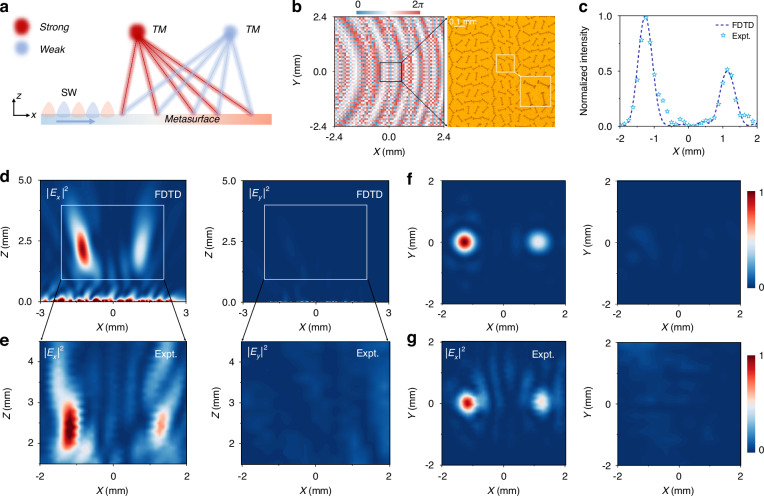
Characterization of dual-focus beams generated by the on-chip MS. **a** Diagram of dual-focus beam radiations with both TM polarization by the on-chip composite MS excited by SW from left-side plasmonic metal. **b** The orientation angle distribution of the entire MS and a top-view image of a section of the fabricated MS, consisting of 60 × 60 meta-units. **c** Simulated and experimental field intensities of the dual focus beams along a line on the focal plane, i.e., *z* = 2.4 mm and *y* = 0 mm. **d**-**g** Simulated and measured ${{{|E}}_{x}|}^{2}$ and ${{{|E}}_{y}|}^{2}$ field distributions in the *xz* plane (*y* = 0 mm) and the *xy* plane (*z* = 2.4 mm) of the composite MS excited by SW. Here, the frequency was fixed at 0.4 THz

Next, the second on-chip MS was demonstrated for generating two focused beams with desired polarizations and intensities (see Fig. 4a), in order to further prove the versatility of the strategy. Following the generic approach described in the section entitled “Fundamental principle”, the target far field was expressed as a linear combination of an LCP beam and an RCP beam, which exhibited different amplitudes and were focused to different focal points. The corresponding NF sources were then retrieved from these far-field beams, based on analytical method rather than computational iterations. The complex fields, featured by two separate focal points with desired intensities and polarizations, were expressed as a superposition of the amplitude-phase distribution {${A}_{{NF}}^{\pm }\left(x,y\right){,\varphi }_{{NF}}^{\pm }\left(x,y\right)$} of the LCP and RCP components:

7 $$\begin{array}{l}\begin{array}{l}\begin{array}{l}{A}_{{NF}}^{+}\left(x,y\right)=\frac{\left|{A}_{1}^{+}{e}^{i{\varphi }_{1}^{+}\left(x,y\right)}+{A}_{2}^{+}{e}^{i{\varphi }_{2}^{+}\left(x,y\right)}\right|}{{A}_{1}^{-}+{A}_{2}^{-}}\\ {\varphi }_{{NF}}^{+}\left(x,y\right)=arg\left({A}_{1}^{+}{e}^{i{\varphi }_{1}^{+}\left(x,y\right)}+{A}_{2}^{+}{e}^{i{\varphi }_{2}^{+}\left(x,y\right)}\right)\end{array}\\ {A}_{{NF}}^{-}\left(x,y\right)=\frac{\left|{A}_{1}^{-}{e}^{i{\varphi }_{1}^{-}\left(x,y\right)}{e}^{i\Delta {\varphi }_{1}}+{A}_{2}^{-}{e}^{i{\varphi }_{2}^{-}\left(x,y\right)}{e}^{i\Delta {\varphi }_{2}}\right|}{{A}_{1}^{-}+{A}_{2}^{-}}\end{array}\\ {\varphi }_{{NF}}^{-}\left(x,y\right)=arg\left({A}_{1}^{-}{e}^{i{\varphi }_{1}^{-}\left(x,y\right)}{e}^{i\Delta {\varphi }_{1}}+{A}_{2}^{-}{e}^{i{\varphi }_{2}^{-}\left(x,y\right)}{e}^{i\Delta {\varphi }_{2}}\right)\end{array}$$

where:

8 $${\varphi }_{i}^{+}\left(x,y\right)={\varphi }_{i}^{-}\left(x,y\right)=-{k}_{0}\cdot \left(\sqrt{{\left(x-{x}_{i}\right)}^{2}{+\left(y-{y}_{i}\right)}^{2}+{F}^{2}}-F\right)$$

represent the standard parabolic phase distribution needed to generate the *i*-th beam focused to the point (${x}_{i}{,y}_{i},F$), being identical for LCP and RCP component. On top of that, a spatially independent phase $\Delta {\varphi }_{i}$ was intentionally added to describe the phase difference between the LCP and RCP components within the focused beam, thereby determining the polarization state of the *i*-th focused beam. Here, $\{{A}_{i}^{+},\,{A}_{{\rm{i}}}^{-}\}$ denote the field amplitudes of the LCP and RCP components for generating the *i*-th focused beam, representing different intensities of two generated beams. Correspondingly, ${A}_{{ms}}^{\pm }\left(x,y\right)$ is normalized by ${A}_{1}^{\pm }+{A}_{2}^{\pm }$. Therefore, the intensities and polarizations of two focused beams can be freely tuned by adjusting $\{{A}_{i}^{+},{A}_{{\rm{i}}}^{-}\}$ and ∆*φ*_*i*_ correspondingly (see more details in the Supplementary Information Section 4).

Setting parameters ${A}_{1}^{\pm }=1$, ${A}_{2}^{\pm }=1/\sqrt{2}$, *x*_1_ = -*x*_2_ = -1.2 mm, *y*_1_ = *y*_2_ = 0 mm, *F* = 2.4 mm and ∆*φ*_1_ = ∆*φ*_2_ = 0, the rotation angle distributions {*θ*_*i*_} of the meta-atomZs were obtained by substituting Eq. (7) to Eq. (4). The THz sample was then fabricated according to the angle distribution, with the sample picture shown in Fig. 4b.

The same near-field characterization system was used to measure the *E*-field distributions (${|{E}_{x}|}^{2}$ and ${|{E}_{y}|}^{2}$) on both the *xz* plane (y = 0 mm) and the *xy* plane (*z* = 2.4 mm) of the meta-device excited by a SW beam at 0.4 THz, as depicted in Fig. 4e, g. FDTD simulations were also performed to verify wave scatterings of the MS under the same SW excitation, yielding results (see Fig. 4d, f) agreeing well with the experiment. The ratio between the field intensities carried by two beams at the focal point was found to be 1:0.52 experimentally and 1:0.50 in the simulation (Fig.4c), both agreeing well with the theoretical prediction (1:0.50).

### High-quality holography by on-chip complex-amplitude metasurfaces

**Fig. 5:**
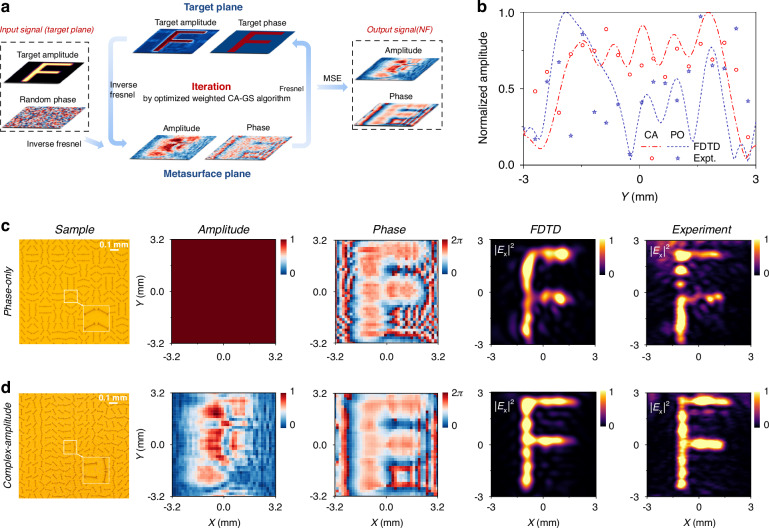
Comparison between PO and CA holograms with scalar linear polarization using different on-chip THz MSs under the SW excitation. **a** Flowchart of the optimized CA GS algorithm for designing holographic MS. **b** Field Intensity comparison between two different holograms at *x* = −1.3 mm in the image plane obtained in **c**, **d**. **c**, **d** Sample images, design parameters, and ${|{E}_{x}|}^{2}$ field intensities obtained by simulations and experiments in the target plane (*z* = 1.5 mm) of the PO and CA MS holograms under the excitation of SW 0.4 THz

Next, the third on-chip CA MS was designed to generate a pre-designed holographic image, showcasing its superior performance compared with its PO counterpart. An optimized iteratively-weighted CA-GS algorithm was first developed, as shown in Fig. 5a, addressing the limitations of the conventional direct-retrieval approach, such as loss of amplitude information and reliance on the paraxial approximation. As an example, a character “F” with a random phase distribution on the target plane was selected as the image. By applying inverse Fresnel diffraction, the initial amplitude and phase distributions $\{{A}_{{NF}}^{\pm }\left(x,y\right){,\varphi }_{{NF}}^{\pm }\left(x,y\right)\}$ on the MS plane were obtained. Fresnel diffraction was then performed to yield the new amplitude and phase distributions $\left\{{A}_{{tar}}^{\pm }\left(x,y\right){,\varphi }_{{tar}}^{\pm }\left(x,y\right)\right\}$ for the target plane. Next, the iteratively retrieved amplitude distribution was replaced by that of the desired holographic image and a weighted element was introduced to accelerate the iterative process. In addition, unlike the conventional GS algorithm, this method includes the phase distribution on the imaging plane in the iteration process to generate the desired phase profile. The iteration was optimized to avoid getting trapped by local minima and the iteration speed was accelerated by incorporating a gradient-descent algorithm (see more details in the Supplementary Information Section 5). Finally, the iteration was terminated when the mean square error (MSE) between the generated image and the target image became smaller than a pre-determined threshold, yielding the amplitude-phase $\{{A}_{{NF}}^{\pm }\left(x,y\right){,\varphi }_{{NF}}^{\pm }\left(x,y\right)\}$ distributions for the meta-device. The amplitude-phase information {${A}_{{NF}}^{+}\left(x,y\right){,\varphi }_{{NF}}^{+}\left(x,y\right)$} and {${A}_{{NF}}^{-}\left(x,y\right){,\varphi }_{{NF}}^{-}\left(x,y\right)$} was encoded within the even and odd columns of the CA MS. Under the condition that the amplitudes and phases of the LCP and RCP components were identical, the phase difference at the target plane was described as:

9 $$\Delta {\varphi }_{{tar}}\left(x,y\right)={\varphi }_{{tar}}^{+}\left(x,y\right)-{\varphi }_{{tar}}^{-}\left(x,y\right)$$

Here, $\Delta {\varphi }_{{tar}}\left(x,y\right)$ is set to zero, where ${\varphi }_{{tar}}^{+}(x,y)$ and ${\varphi }_{{tar}}^{-}\left(x,y\right)$ denote the phase distributions at the target plane for two orthogonal polarizations. Therefore, it can be predicted that the interference between these two components resulted in a holographic image with a homogeneous linear polarization. By substituting the obtained amplitude and phase distribution {${A}_{{NF}}^{\pm }\left(x,y\right){,\varphi }_{{NF}}^{\pm }\left(x,y\right)$} of the MS plane into Eq. (4), the rotation angles {*θ*_*i*_} for each meta-atom of the desired MS were retrieved, and the meta-device was then fabricated together with the eigen-SW plate.

THz near-field scanning experiments were performed to characterize the CA holographic effect of the fabricated on-chip CA meta-device. Figure 5d shows the image of a portion of the fabricated MS sample, consisting of 80 × 80 meta-atoms with specially designed rotation angles. Figure 5d also depicts the simulated and measured ${|{E}_{x}|}^{2}$ patterns on the *xy* plane at $z$ = 1.5 mm above the MS, under the excitation of a SW beam at 0.4 THz launched by a meta-coupler placed on the left-hand side of the sample. For comparison, another MS based on PO modulation was also fabricated to generate the same holographic image. A portion of the fabricated PO-MS sample is shown in Fig. 5c. Both experimental measurements and numerical simulations were conducted under identical SW excitation conditions to evaluate their holographic performance. Quantitative comparison based on the mean square error (MSE) of the reconstructed holograms reveals values of 0.1752 (PO-MS) and 0.078 (CA-MS) (see more details in the Supplementary Information Section 6). To further illustrate the high quality of the reconstructed holographic image, Fig. 5b presents the measured electric field distribution of the generated CA holography along the *x* = −1.3 mm line on the image plane, showing excellent field uniformity. In addition, iterative application of the present algorithm allows additional control over the phase distribution at the target plane, resulting in a more uniform phase profile in CA holography compared with its PO counterpart. The simulated and experimental phase distributions at the target plane were provided in the Supplementary Information Section 7. These results clearly demonstrate the superior quality of the reconstructed holographic image by the CA-MS, which benefits from its enhanced abilities to independently control both the magnitude and direction of the wave-vector components, compared with its PO counterpart. Moreover, owing to the broadband characteristics of the SW decoupling mechanism^34^, the generated CA holography maintains stable performance over a bandwidth of approximately 0.1 THz (see the Supplementary Information Section 8 for details).

### Vectorial complex-amplitude coding meta-holography by on-chip metasurface

**Fig. 6:**
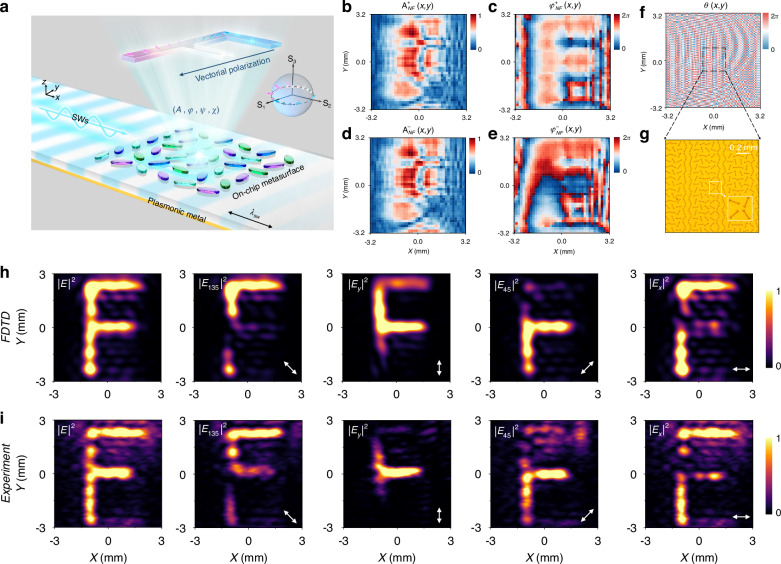
The CA vectorial holography generated by the on-chip composite MS under SW excitation using the optimization algorithm. **a** Diagram of CA vectorial hologram by the on-chip MS under SW excitation. **b**-**e** Amplitude and phase distribution of LCP **b**, **c** and RCP **d**, **e** components emitted by the on-chip CA MS. **f**-**g** Orientation angle distribution of entire MS and partial image of the fabricated sample. **h**-**i** Simulated and measured electric field intensities projected to different linear polarization directions (135° ,90°, 45°, 0°) of the generated vectorial CA hologram in *xy* plane (*z* = 1.5 mm) at 0.4 THz

Finally, the fourth SW-excited MS was demonstrated to generate a *vectorial* holographic image on the target plane exhibiting spatially varying polarization (see Fig. 6a). Following the generic design approach described in the previous section, the target vectorial image was first decomposed into two holographic images carrying LCP and RCP, respectively, exhibiting specific amplitude and phase distributions, as described by Eq. (9). In contrast with the scalar holography, the CA-GS algorithm was employed to perform a two-loop iterative process. This approach was used to retrieve the amplitude–phase distributions of the LCP and RCP components required for constructing the MS, under the constraint on their phase difference ($\Delta {\varphi }_{{tar}}\left(x,y\right)={\varphi }_{{tar}}^{+}\left(x,y\right)-{\varphi }_{{tar}}^{-}\left(x,y\right)=2\pi \cdot \frac{(-y+3.2\mathrm{mm})}{6.4\mathrm{mm}}$). By retrieving the amplitude-phase distributions of the LCP component {${A}_{{NF}}^{+}\left(x,y\right){,\varphi }_{{NF}}^{+}\left(x,y\right)$}(Fig. 6b, c) and the RCP component {${A}_{{NF}}^{-}\left(x,y\right){,\varphi }_{{NF}}^{-}\left(x,y\right)$}(Fig. 6d, e), the entire rotation-angle distributions {*θ*_*i*_} of the meta-atoms were then retrieved from Eq. (4) to construct the desired MS, as shown in Fig. 6f. Based on these retrieved {*θ*_*i*_} distributions, the MS was fabricated by arranging the meta-atoms accordingly. An optical image of a portion of the fabricated sample is shown in Fig. 6g.

THz near-field scanning measurements were then performed to characterize the functionality of the fabricated meta-device, again under the excitation of a SW at 0.4 THz launched by a meta-coupler. Figure 6h, i shows the simulated and measured *E-*field intensity distributions on the image plane *z* = 1.5 mm above the MS, which are consistent with each other, both showing a clear character “F”. The vectorial nature of the generated image was characterized to measure its different field components by rotating the THz MS along four directions (135°, 90°, 45°, 0°) while keeping the probe fixed. The resulting patterns are presented in the remaining panels of Fig. 6i. These measured patterns, in good agreement with corresponding simulated patterns under the same conditions, demonstrate clearly that the generated pattern is a vectorial one with polarization distribution consistent with the theoretical design, as indicated by the arrows in Fig. 6a. Generally, the spatial resolution of scalar or vectorial holographic image is fundamentally proportional to the pixel size and total pixel number of the metahologram. By increasing these two values while adhering to the Nyquist sampling criterion, the overall image quality can be further improved.

## Discussion

In summary, a general strategy for designing the composite on-chip CA MS that can generate scalar or vectorial radiation fields under SW excitation is proposed and demonstrated. The building block, consisting of 2 × 2 PB meta-atoms for generating both LCP and RCP components, gives complete control over all the EM characteristics (including amplitude, phase, polarization) of local radiation fields at subwavelength level, relying on their internal interference effect. As proof of concept, several on-chip SW-driven MSs working in the THz regime were designed and fabricated to achieve dual beam generation, dual focusing, and meta-holography with specific homogeneous polarization. Moreover, by introducing the spatially varying phase difference on the imaging plane for LCP and RCP radiation components, an on-chip CA MS capable of generating vectorial holography was achieved. The NF measurements and FDTD simulations verify the theoretical prediction. Compared with the conventional on-chip devices, the MS exhibits extraordinary wave-control capabilities through fully utilizing all EM characteristics. The scheme is quite general and can be extended to other frequency regimes such as the visible and microwave bands (see more details in the Supplementary Information Section 9). Additionally, the approach is applicable to the generation of vectorial vortex fields (see more details in the Supplementary Information Section 10), and other forms of CA vectorial holographic images (see more details in the Supplementary Information Section 11). These results establish a novel ultra-compact on-chip platform for generating arbitrary structured fields, paving the way for advanced optical applications in super-resolution imaging, augmented reality, and integrated optics.

## Materials and methods

### Fabrication

The fabrication of THz sample was carried out through three main steps: preparation of the decoupling MS, fabrication of the coupling MS, and alignment of the two parts. The decoupling MS was patterned on a 55-μm-thick quartz substrate through photolithography, gold deposition, and lift-off processes performed on both sides of the substrate. The coupling MS was fabricated on a second quartz substrate using the same photolithography and thin-film deposition procedures. Finally, the two substrates were precisely aligned and bonded with UV adhesive to form the complete THz MS device. The detailed fabrication procedure is provided in the Supplementary Information Section 1.

### Numerical simulations

Finite-difference time-domain (FDTD) simulations were performed to demonstrate the performance of the devices. The reflection properties of the meta-atoms were calculated by applying unit-cell boundary conditions in the *x*- and *y-*directions and two ports in the *z* direction as the source and receiver. In the calculations, the relative permittivity of quartz was set as $3.9+0.001i$, while gold (Au) was modeled with a conductivity of 5 × 10^7^ S∙m^−1^. To demonstrate the scalar or vectorial radiation fields generated by the on-chip CA MS, open boundary conditions were adopted around the device to absorb the energy of all outgoing radiation fields. The full complex electric fields, including both the amplitude and phase information, were extracted from the FDTD results and used to verify the intensity and polarization characteristics of the radiated fields.

## Supplementary information

Supplemental Material

## Data Availability

All key data that support the findings of this study are included in the article and its Supplementary Information. Additional datasets and raw measurements are available from the corresponding authors upon reasonable request.
