# Supplementary material for: Generating vectorial optical fields via surface-wave-excited complex-amplitude metasurfaces
Source: Light Sci Appl. 2026 May 27;15:256. doi: 10.1038/s41377-026-02334-1 (PMC13216528; doi:10.1038/s41377-026-02334-1)
Supplement: Supplementary file 1 — Supplemental Material [file 41377_2026_2334_MOESM1_ESM.docx]

**Supplementary Information for: Generating Vectorial Optical Fields via Surface-Wave-Excited Complex-Amplitude Metasurfaces**

*Xiangyu Jin^1#^, Yu He^2#^, Jianru Li^1^, Xiaoya Nie^1^, Shuai Du^1^, Yufei Song^2^, Haoyu Luo^1^, Muhan Liu^1^, Shaojie Ma^1,3^, Qiong He^2,3^, Lei Zhou^2,3*^, Zhuo Wang^3,4*^, Shulin Sun^1,3*^*

*^1^Shanghai Engineering Research Centre of Ultra Precision Optical Manufacturing, Department of Optical Science and Engineering, College of Future Information Technology, Fudan University, Shanghai 200433, China*

*^2^State Key Laboratory of Surface Physics, Key Laboratory of Micro and Nano Photonic Structures (Ministry of Education) and Department of Physics, Fudan University, Shanghai 200433, China*

*^3^Shanghai Key Laboratory of Metasurfaces for Light Manipulation, Shanghai 200433, China*

*^4^Shanghai Frontiers Science Research Base of Intelligent Optoelectronics and Perception, Institute of Optoelectronics, Fudan University, Shanghai 200433, China.*

Keywords: Complex amplitude; Vectorial holography; On-chip metasurface; Terahertz; Surface wave

# S1. Fabrication process of the terahertz metasurface sample

The fabrication of the THz sample was carried out through three main steps: (i) preparation of the meta-coupler for SW excitation, (ii) fabrication of the plasmonic metal and meta-decoupler for transporting and decoupling SW, and (iii) substrate alignment and bonding. As illustrated in Fig. S1a, the sample was realized on a 55-μm-thick quartz substrate, which was first patterned by photolithography (MA6), followed by the deposition of a 100-nm-thick gold film using magnetron sputtering (DE500). A standard lift-off process with acetone was then employed to remove the excess metal and retain the designed patterns. Subsequently, aligned photolithography was performed on the back face of the same substrate, followed by additional thin-film deposition and lift-off steps. The resulting SW-excitation MS is shown in Fig. S1b, with air-holes radius of 60 μm. For the SW-decoupling MS, a second 55-μm-thick quartz substrate was processed via photolithography and thin-film deposition to create the meta-decoupler and plasmonic metal sample, as shown in Fig. S1c. Finally, the two quartz substrates were carefully aligned and bonded together with UV adhesive, yielding the complete THz MS device.


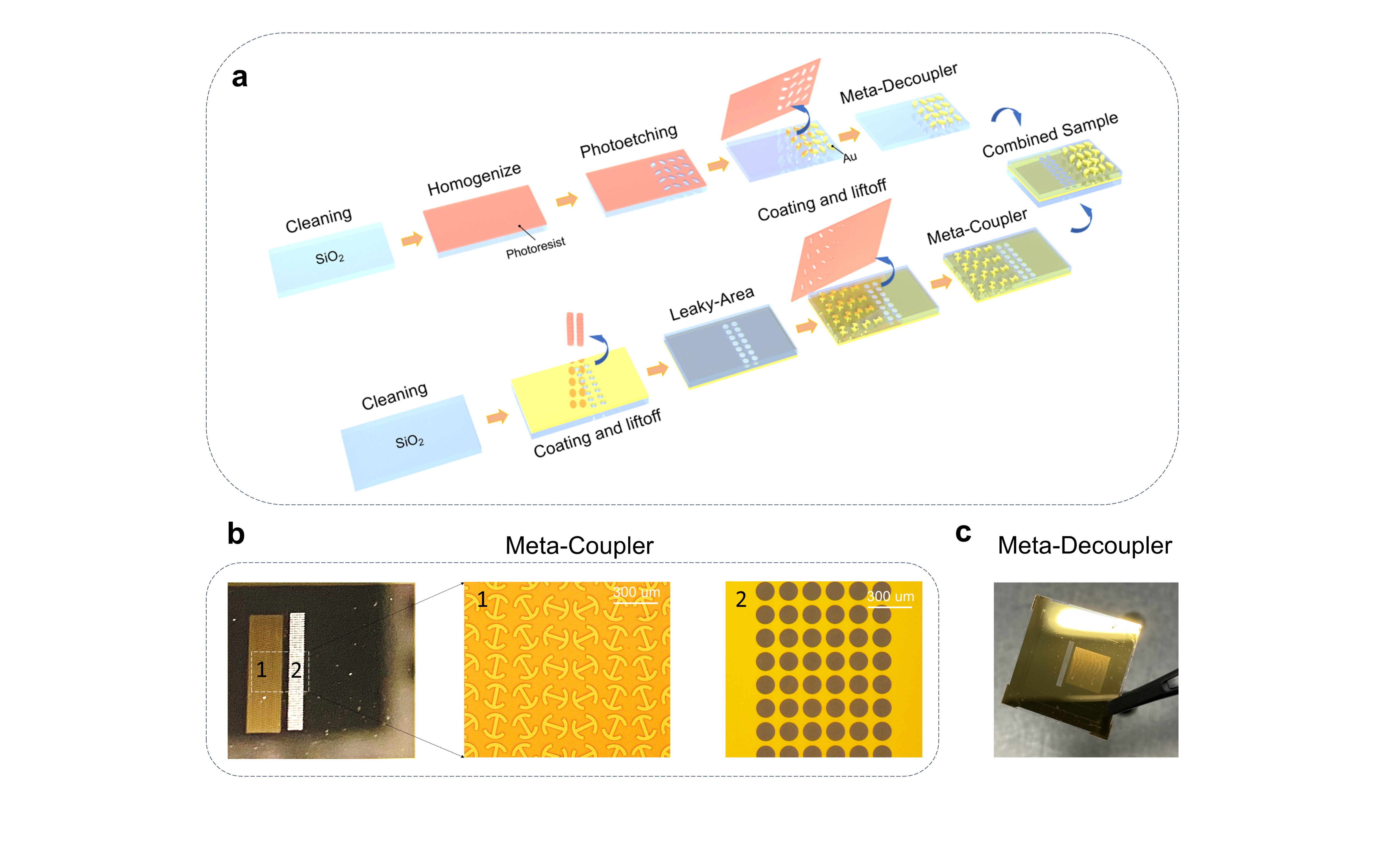


**FIG. S1. Fabrication process of the whole sample, consisting of a SW meta-coupler, plasmonic metal and SW meta-decoupler.** (a) Fabrication process of the whole sample. (b-c) Meta-coupler and meta-decoupler of the fabricated sample.

# S2. Design and characterization of the terahertz meta-coupler for exciting surface wave


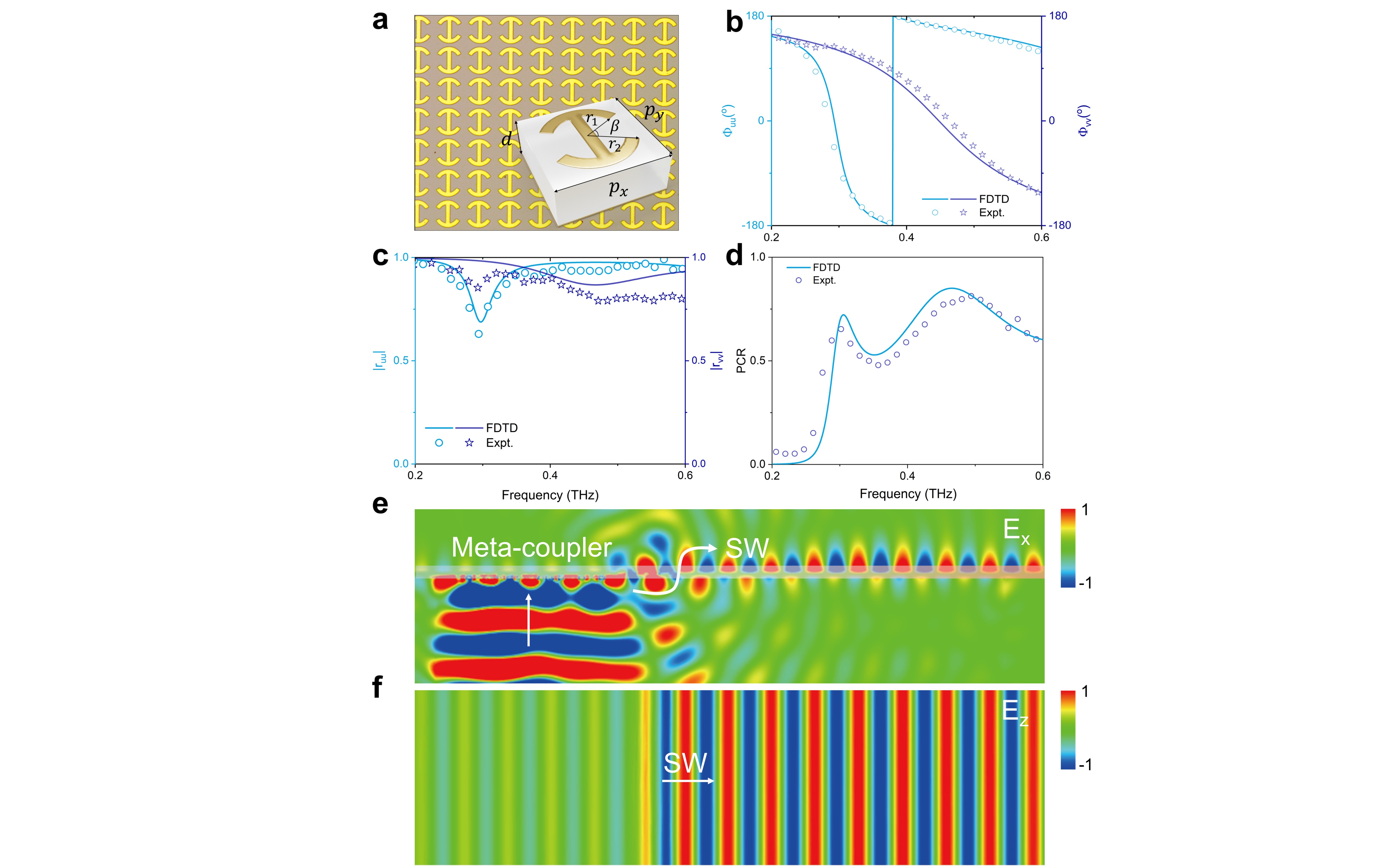


**FIG. S2. Characterization of the meta-coupler for launching SW on the plasmonic metal.** (a) Diagram of the fabricated PB meta-atoms composed of curved I-shaped metallic microstructures as the top layer and a metallic thin film as the bottom layer, separated by a 55-μm thick dielectric spacer ($\varepsilon_{r}=3.9+0.001i$). The inset figure shows the geometry of the meta-atom with the parameters of $p_{x}=p_{y}=160$μm, $d=$55 μm, $r_{1}=70$μm, $r_{2}=50$ μm, $\beta={60}^{^{\circ}}$. (b-d) Spectra of the reflection phase ($\Phi_{uu}$ and$\Phi_{vv}$), reflection amplitude (${|r}_{uu}|$ and $|r_{vv}|$), and polarization conversion ratio (PCR) of the periodic PB meta-atoms. (e, f) Simulated $E_{x}$ and $E_{z}$ distributions inside the meta-coupler for SW excitation on the $xz$plane ($y =$0 mm) and the $xy$ plane at ($z=$0 mm).

# S3. Terahertz time-domain near-field spectroscopy system

A broadband terahertz beam emitted from the laser was first expanded and collimated using a lens. After that, its polarization state was converted to circular polarization via the combination of a linear polarizer (LP) and a half-wave plate (HWP). Free-space circularly polarized beam was then coupled into surface waves by the meta-coupler and then decoupled back to the desired structured light by the metasurface (MS). Finally, the waves emitted by the MS were detected using a terahertz probe.


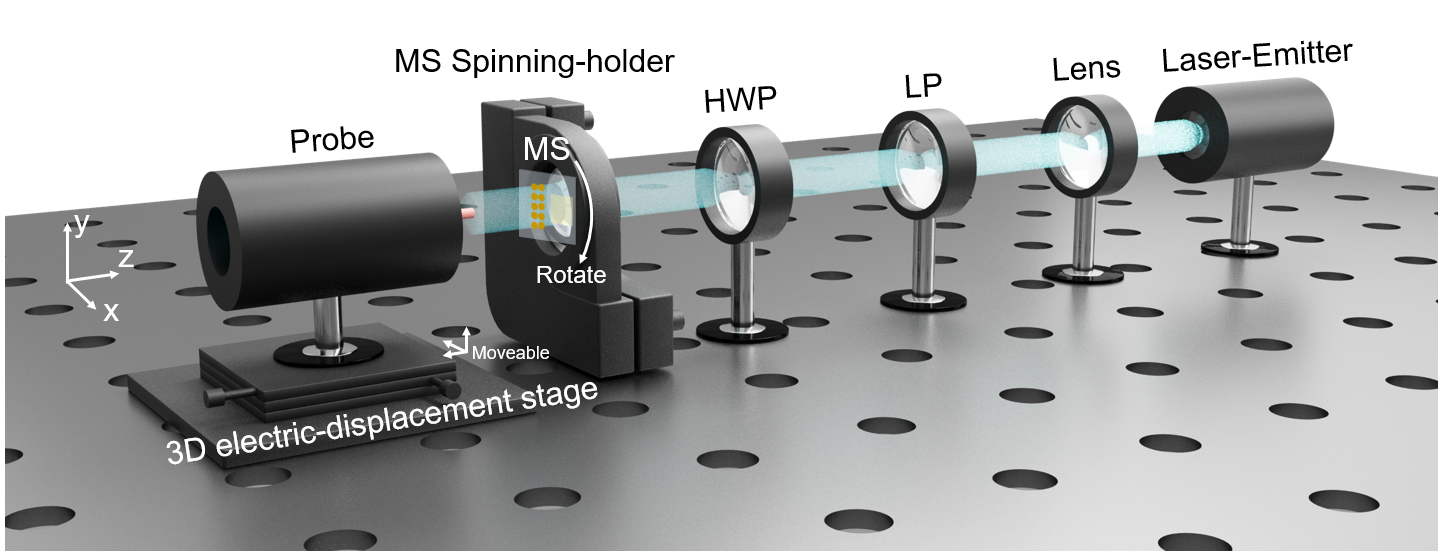


**FIG. S3. Diagram of the near-field measurement setup for mapping the electric field pattern (including ampltidue and phase) emitted by the SW-excited metasurfaces.**

# S4. Dual focal beams with the arbitrary intensity and polarization states

$$\tilde{E}_{odd}^{+}\left( x,y \right)={(A}_{1}^{+}e^{i\varphi_{1}^{+}\left( x,y \right)}+A_{2}^{+}e^{i\varphi_{1}^{+}\left( x,y \right)})e^{-i\varphi_{sw}^{+}}$$

$$\tilde{E}_{even}^{-}\left( x,y \right)={(A}_{1}^{-}e^{i\varphi_{1}^{-}\left( x,y \right)}e^{i\Delta\varphi_{1}}+A_{2}^{-}e^{i\varphi_{1}^{-}\left( x,y \right)}e^{i\Delta\varphi_{2}})e^{-i\varphi_{sw}^{-}}$$

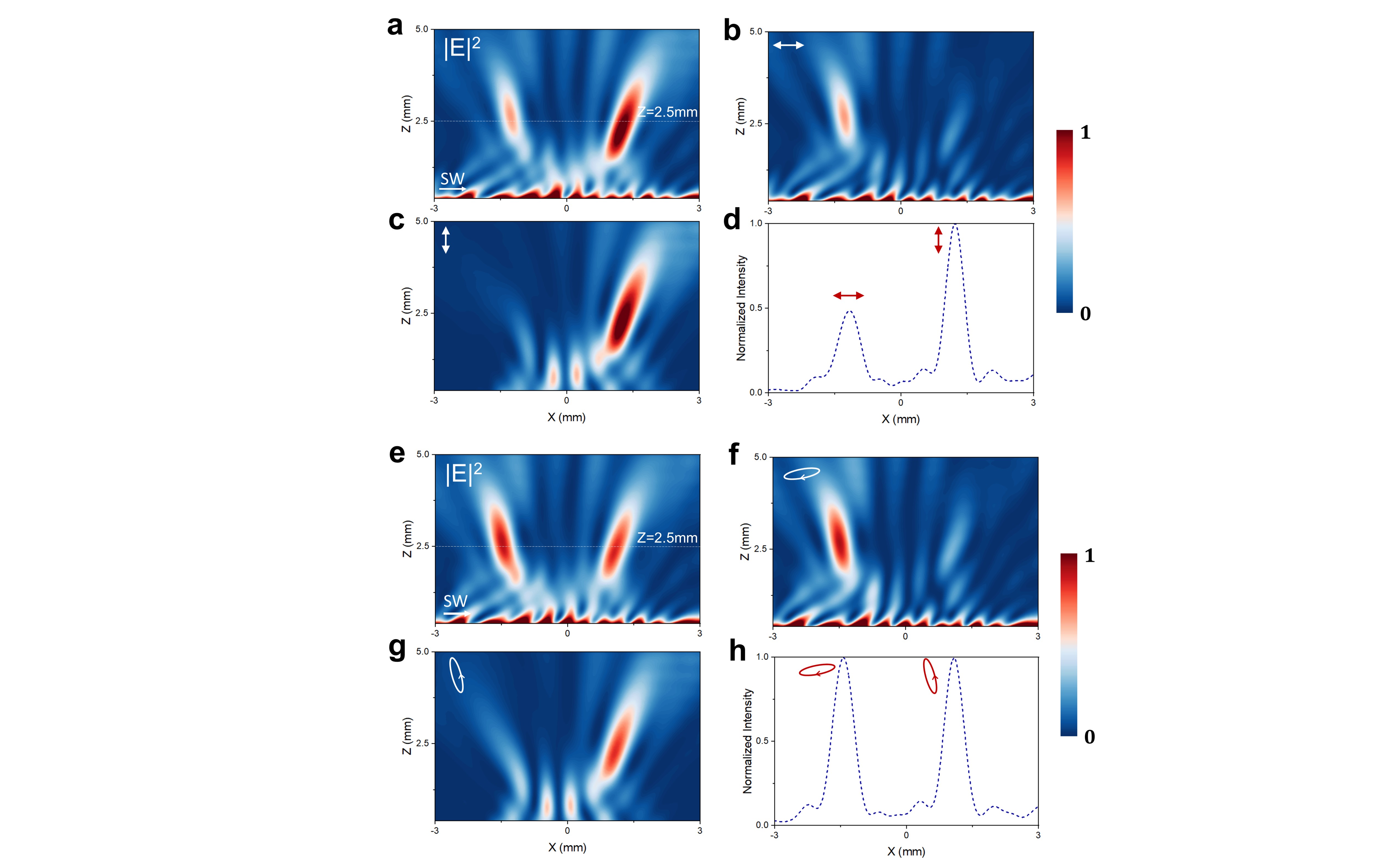


**FIG. S4. Dual focus beams with the arbitrary intensity and polarization states generated by the on-chip metasurface.** (a-d) Dual focus beams generation with the different intensities (0.5:1) and the orthogonal linear polarizations (TM-, TE-linear polarizations). (e-h) Dual focus beams generation with the same intensities but the different elliptical polarizations. Here, $A_{2}^{+}/A_{1}^{+}=1/\sqrt{2}$, $A_{2}^{-}/A_{1}^{-}=\sqrt{2}/1$, $\Delta\varphi_{1}=0$, $\Delta\varphi_{2}=\pi$ were set to achieve this goal.

# S5. Comparison between the traditional GS and modified GS algorithm

The traditional Gerchberg–Saxton (GS) algorithm is the phase retrieval method for designing the MS to achieve the desired complex wavefront distribution in the image plane. As shown in Fig. S5a, the target holographic image on the image plane is defined. Its amplitude distribution is derived from that of the target holographic image, while its phase profile is randomly initialized. The algorithm then iteratively performs forward and inverse Fourier transforms between the MS and image planes. During each iteration, while the phase information on the MS plane is preserved in the structural design, the amplitude information, which contains the inhomogeneous spatial distribution, has to be uniformly set to unity due to the lack of freedom in the design of most metaholograms. Eventually, the phase information can be obtained to design the phase-only (PO) MS. However, the quality of the reconstructed holographic image by such a PO MS is degraded.

In this work, a modified weighted complex-amplitude (CA) GS algorithm is proposed for generating the vectorial holography through simultaneously and independently controlling both the phase and amplitude of the LCP and RCP fields radiated by the MS. As shown in the flowchart in Fig. S5b, the algorithm starts by generating a target field “F” on the image plane with a randomly initialized phase distribution. The iteration performs Fresnel diffraction from the MS plane to the image plane, where the amplitude is replaced with the desired target image “F”. To accelerate the convergence of this method, weighting factors $w_{n}$are introduced to assign different importance to the various regions of the light field. The weight factors for the $n^{th}$ iteration are defined as:

$$A_{n}=A_{n-1}\times w_{n-1}$$

$w_{n}={|A}_{tar}/A_{n}|\times w_{n-1}$.

Here, $A_{n}$ is the $n^{th}$amplitude distribution on the image plane, and $A_{tar}$is the amplitude of the target holographic image. Additionally, the phase distribution on the image plane can be arbitrarily manipulated to be either uniform or inhomogeneous. Unlike the traditional GS algorithm, the amplitude distribution will also be iteratively updated instead of always being set to unity during each iteration. The iteration process will continuously run until the Mean Squared Error (MSE) of both the amplitude and phase distributions of the target image reaches a predetermined threshold. When the iteration process terminates, it yields the final complex amplitude distributions on the MS plane. Meanwhile, to prevent the iterative algorithm from dropping to the local minimum solution, optimization steps are often introduced using a gradient descent algorithm or genetic algorithm.


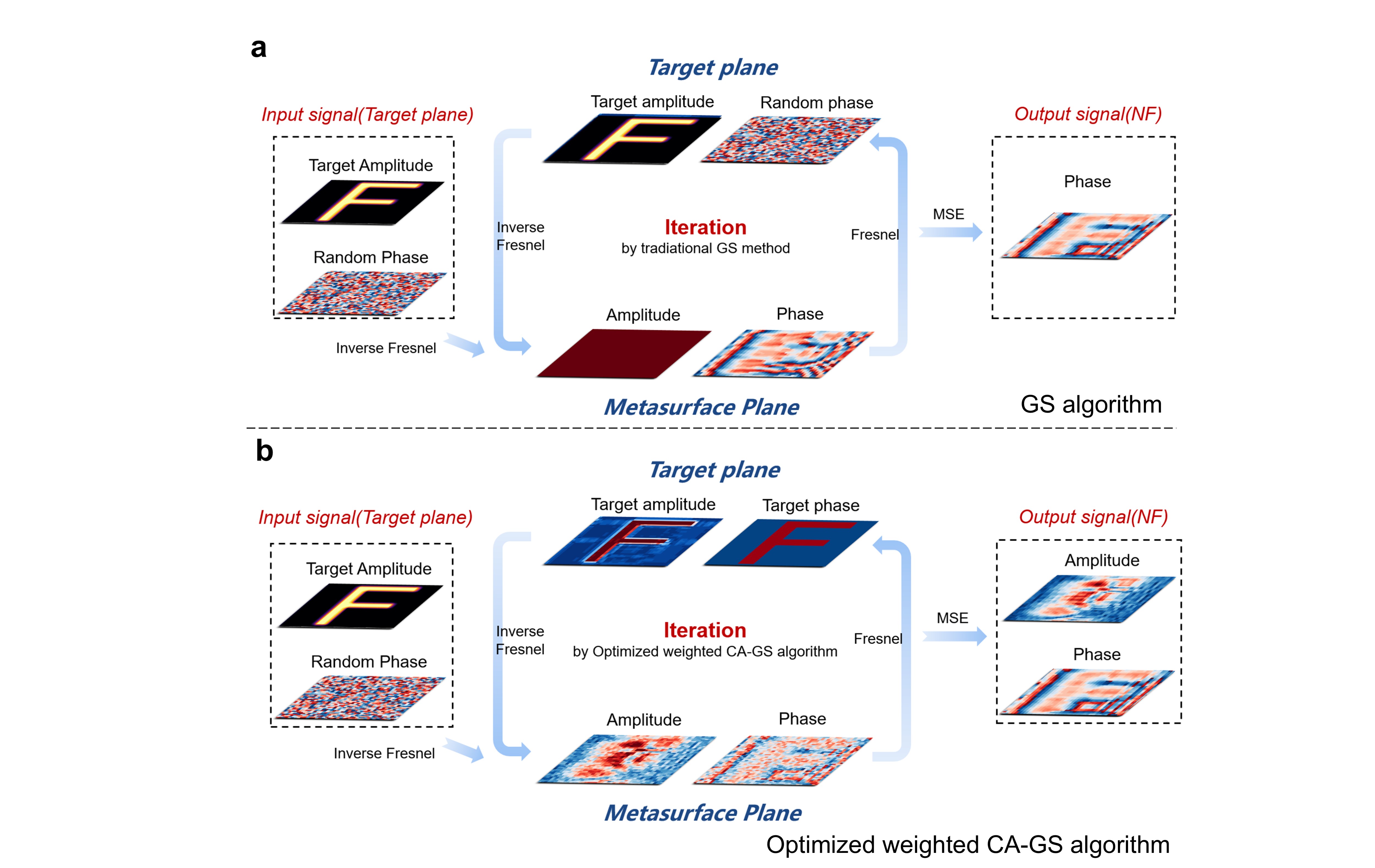


**FIG. S5. Comparison between two GS algorithms.** (a) Flowchart of the traditional GS algorithm for generating the scalar phase-only holography. (b) Flowchart of the modified complex amplitude GS algorithm for generating the complex-amplitude vectorial holography.

# S6. MSE comparison of the phase-only and complex-amplitude holography

The Mean Squared Error (MSE) is widely utilized to evaluate the quality of a generated image, which is defined as:

$$MSE=\frac{1}{MN}\sum_{i=1}^{M} \sum_{j=1}^{N} \left[ I\left( i,j \right)-K(i,j) \right]^{2}$$

Here, *M* and *N* represent the numbers of collected pixels in the image plane along two orthogonal directions. $I(i, j\boldsymbol{)}$ and $K\left( i, j \right)$ represent the complex amplitude (amplitude and phase) distribution of the pre-designed target image and the ultimate generated image with the desired phase at the position labeled as $(i,j)$ on the target plane. The formula evaluates the difference between the theoretical design (amplitude and phase) and the practical realization of the holography.

# S7. Phase distribution of the target plane in the phase-only and complex-amplitude holography


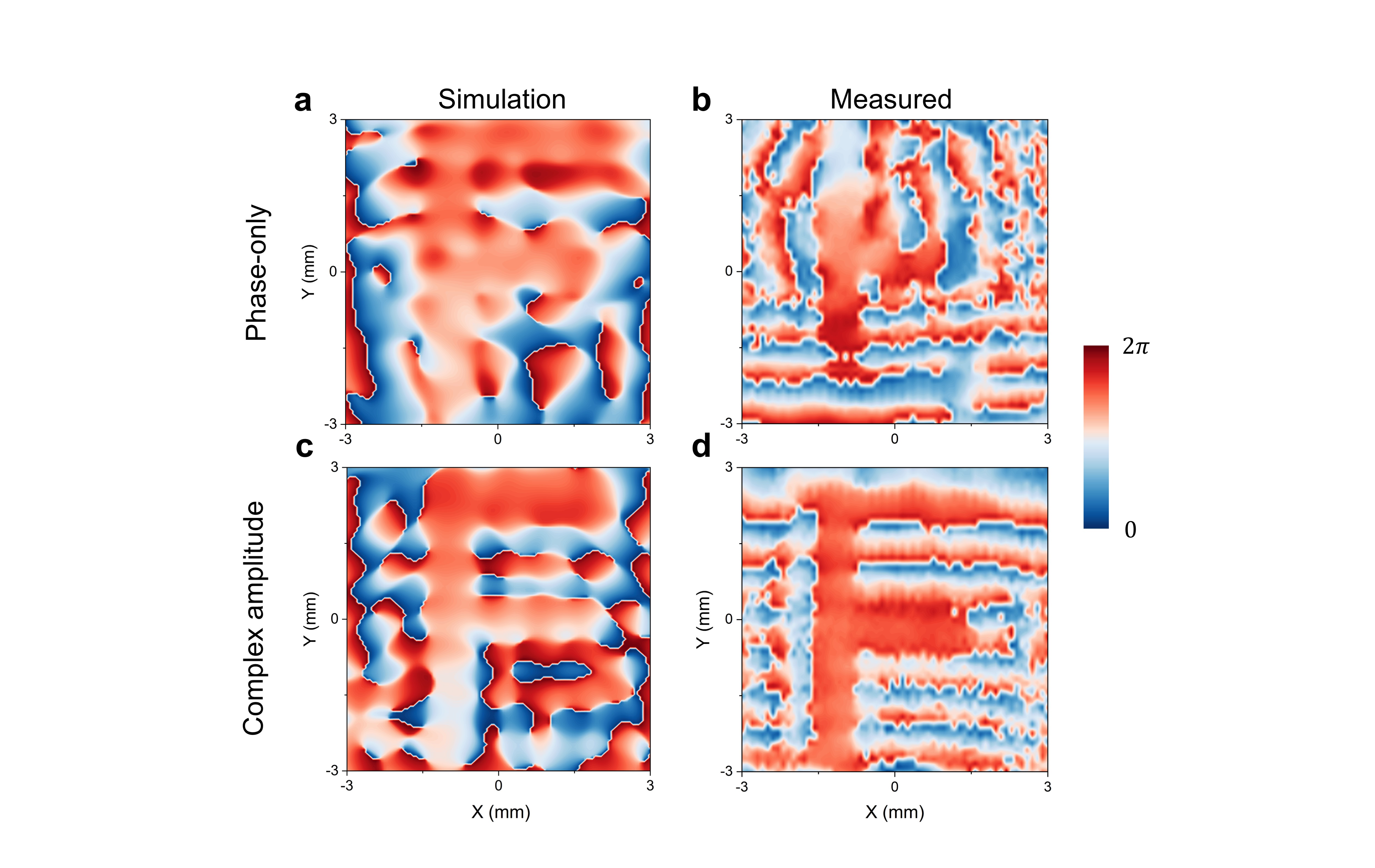


**FIG. S6. Phase distribution of the target image plane (**$\boldsymbol{z}\mathbf{=1.5}$**mm) in phase-only (PO) and complex-amplitude (CA) holography at 0.4 THz.** (a-d) Simulated and measured phase distributions of the PO (a, b) and CA holography (c, d) at the target plane by the designed MS under a SW excitation.

# S8. Broadband performance of the complex amplitude hologram generations


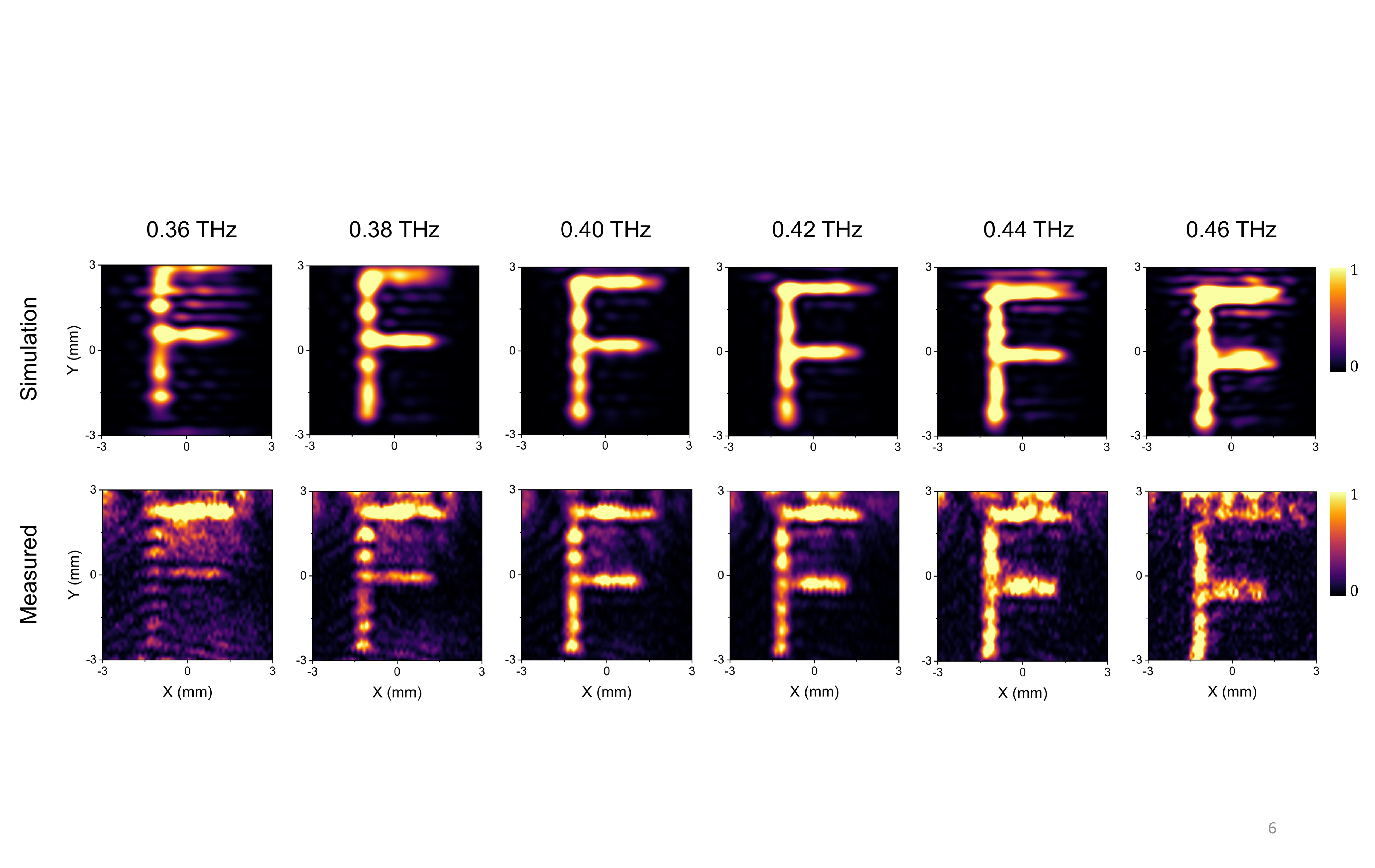


**FIG. S7. Simulated and measured** $\left| \boldsymbol{E}_{\boldsymbol{x}}^{\boldsymbol{2}} \right|$ **distribution of the generated** **hologram at target image plane by the designed MS under SW excitation at 0.36-0.46 THz.**

# S9. Dual-focusing beam generation in the microwave and visible band

The meta-atom working in the microwave regime was designed by simply enlarging the geometric parameters of the THz meta-atom in a metal–insulator–metal (MIM) configuration, as shown in Fig. S8a. The top metallic curved-I pattern has the following geometric parameters: $p=3$mm, $r_{1}=1.33$mm, $r_{2}=1.14$ mm, $w=0.19$ mm and $\alpha={145}^{^{\circ}}$. The dielectric spacer is a $d=2$ mm thick F4B film ($\varepsilon_{r}=3)$. The top and bottom metallic layers can be treated as perfect electric conductor (PEC). Here, the following parameters are chosen for the numerical calculations:$A_{1}^{\pm}=1$, $A_{2}^{\pm}=1/\sqrt{2}$, $x_{1}=-x_{2}=-120$mm, $y_{1}=y_{2}=0$mm, $F=240$mm and $\Delta\varphi_{1}=\Delta\varphi_{2}=0$. Fig. S8b shows the simulated electric field intensity distributions of the complex-amplitude metasurface excited by an impinging surface wave beam at 12 GHz. The intensity ratio of the two focal beams is approximately 1:0.51, in good agreement with the theoretical value of 1:0.50.

For the visible band, dual focal beam generation with a predefined intensity ratio was numerically demonstrated. The basic meta-atom working at around 700 nm wavelength, was designed to consist of a 20 nm thick silver (Ag) nano-brick and 80 nm thick continuous Ag film, separated by a 70 nm thick dielectric spacer ($\varepsilon_{r}=2.25$), as shown in Fig. S8c. The structure parameters are$p=110$nm, $l_{u}=30$nm,$l_{v}=50$nm,$h_{0}=50$nm,$h_{1}=70$nm,$h_{2}=80$nm. The permittivity of silver is described by the Drude model with the plasmon angular frequency $\omega_{p}=$1.37×10^16^ rad/s and damping angular frequency $\omega_{\gamma}=$8.5×10^13^ rad/s. Similar to the designs at other frequencies, the following parameters are chosen: $A_{1}^{\pm}=$1, $A_{2}^{\pm}=1/\sqrt{2}$, $x_{1}=-x_{2}=-$2.2 μm, $y_{1}=y_{2}=0$μm, $F=$4.4 μm and $\Delta\varphi_{1}=\Delta\varphi_{2}=0$. Fig. S8d shows the dual focal field distribution generated by the optical metasurface excited by the surface wave at 700 nm wavelength. The simulated field intensity ratio (approximately 1:0.47) shows good agreement with the theoretical value (1:0.50).


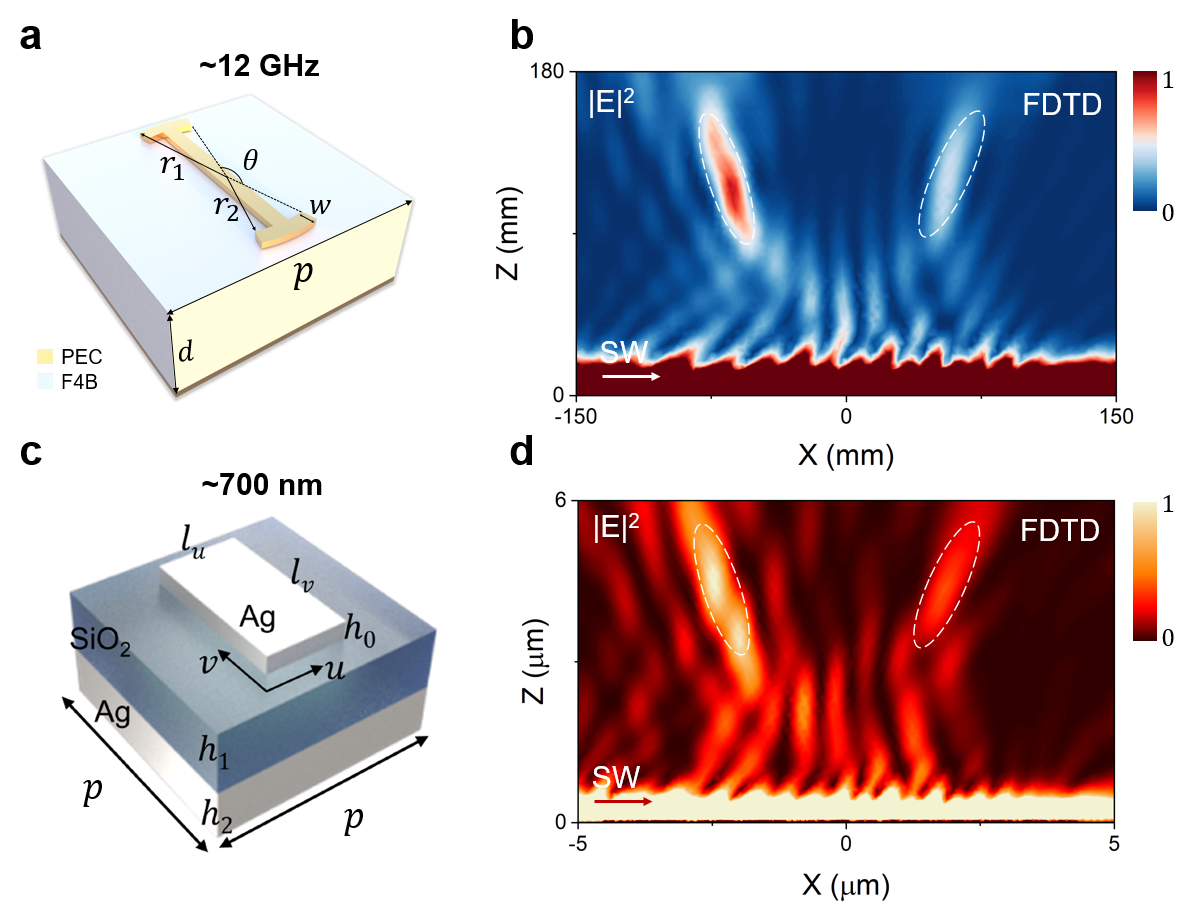


**FIG. S8.** **Characterization of dual-focus beams generated by the on-chip metasurface in the microwave and visible frequency regimes.** (a) Diagram of the anisotropic meta-atom designed in the microwave regime. (b) Simulated ${|E|}^{2}$field distribution in the $xz$ plane ($y = 0$ mm) of the composite MS excited by SW at 12 GHz. (c) Diagram of the anisotropic meta-atom designed in the visible regime. (d) Simulated ${|E|}^{2}$field distribution in the $xz$ plane ($y = 0$mm) of the composite MS excited by SW at a wavelength of 700 nm.

# S10. Vectorial vortex beam generation

The strategy of using the complex amplitude metasurface is quite general and can be developed to construct other vectorial fields, such as vectorial vortex fields carrying orbital angular momentum (OAM), as shown in Fig. S9a. Full-wave simulations were performed to design a metasurface encoded with a focusing phase and a helical phase in the two polarization channels:

$$A_{NF}^{+}\left( x,y \right)=1$$

$$A_{NF}^{-}\left( x,y \right)=1$$

$\varphi_{NF}^{+}\left( x,y \right)=-k_{0}\left( \sqrt{x^{2}+y^{2}+F^{2}}-F \right)+l^{+}$arctan$(y/x)$

$\varphi_{NF}^{-}\left( x,y \right)=-k_{0}\left( \sqrt{x^{2}+y^{2}+F^{2}}-F \right)+l^{-}$arctan$(y/x)$

Here, $F=$3 mm and $l^{+}=-l^{-}=$ 2 denote the focal length and topological charges, respectively. Based on the amplitude-phase distributions of the MS as shown in Fig. S9b, the rotation angle distributions of the meta-atoms were retrieved (see Fig. S9c) by taking the equations into Eq. (4) in the main text. Based on the meta-atom described in the main text, a terahertz metasurface consisting of 80$\times$80 unit cells was designed. FDTD simulations were used to obtain the electric field distributions of the generated vectorial vortex beam, as shown in Fig. S9d. The total electric field (${|E|}^{2}$) pattern in the $xy$plane $(z=$3 mm) reveals the donut-shaped field pattern, representing one clear feature of a vortex beam. Moreover, the electric field patterns of the generated vortex beam, which are projected onto the different linear polarization components respectively, confirm the inhomogeneous polarization feature and its topological charge ($l=\frac{l^{+}+\left| l^{-} \right|}{2}=2$).


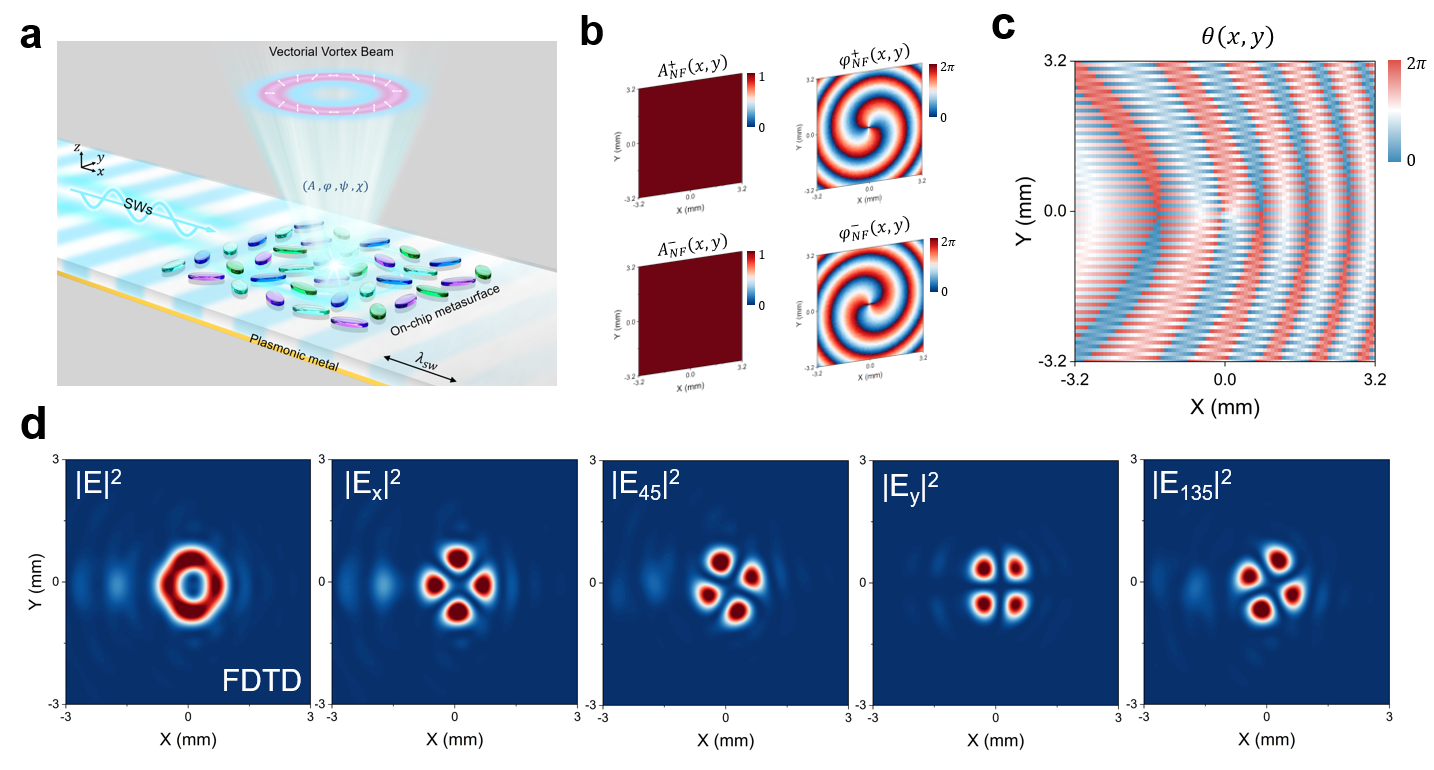


**FIG. S9. Generation of the vectorial vortex beam based on a complex-amplitude on-chip metasurface.** (a) Diagram of the vectorial vortex beam generation. (b) Complex-amplitude distributions of the LCP and RCP components encoded on the metasurface. (c) Retrieved rotation-angle distribution of the meta-atoms. (d) Simulated total electric field and electric field component (projected to different linear polarization directions) distributions of the vectorial vortex beam in $xy$plane $(z=3$mm) generated by the on-chip metasurface.

# S11. Discretized complex-amplitude vectorial holography


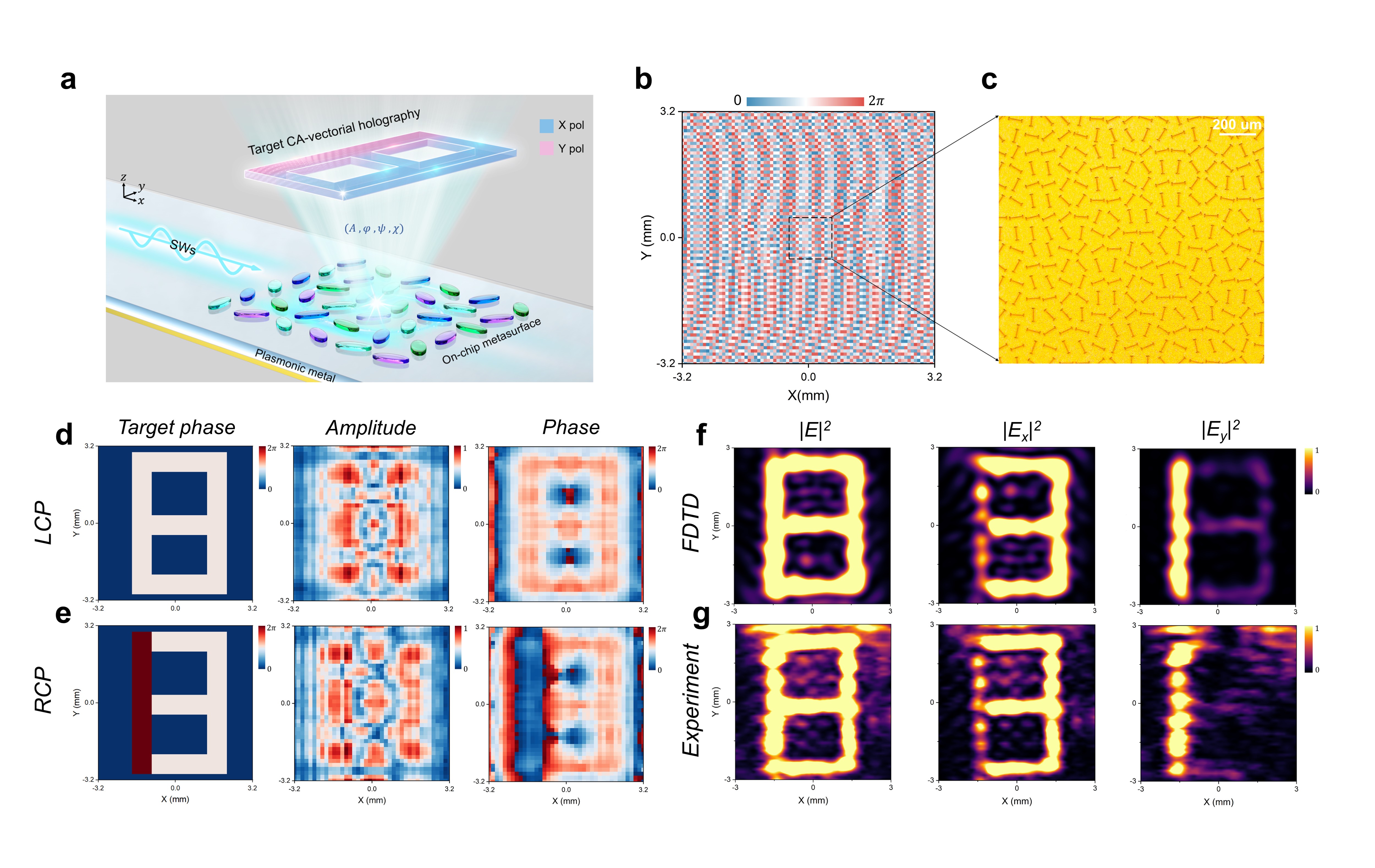


**FIG. S10. The discretized complex amplitude vectorial holography generated by an on-chip composite MS under the SW excitation using the modified two-loop optimization algorithm.** (a) Diagram of the complex amplitude hologram generation with the discretized linear polarizations by the on-chip MS under the SW excitation using the two-loop optimization algorithm. (b-c) The orientation angle distribution of the entire MS and partial image of the fabricated MS sample. (d-e) The amplitude and phase distribution of the LCP and RCP components generated by on-chip MS. (f-g) Simulated and measured field intensities (${|E|}^{2},{|E_{x}|}^{2}, {|E_{y}|}^{2}$) of the discretized CA vectorial hologram in $xy$plane $(z=1.5$mm) generated by the MS under the SW excitation. Here, the frequency is 0.4 THz.
